# Dynamic Changes of Neuroskeletal Proteins in DRGs Underlie Impaired Axonal Maturation and Progressive Axonal Degeneration in Type 1 Diabetes

**DOI:** 10.1155/2009/793281

**Published:** 2009-10-12

**Authors:** Hideki Kamiya, Weixian Zhang, Anders A. F. Sima

**Affiliations:** ^1^Department of Pathology, School of Medicine, Wayne State University, Detroit, MI 48201, USA; ^2^Department of Endocrinology and Diabetes, Graduate School of Medicine, Nagoya University, Nagoya 466-8550, Japan; ^3^Department of Neurology, School of Medicine, Wayne State University, Detroit, MI 48201, USA

## Abstract

We investigated mechanisms underlying progressive axonal dysfunction and structural deficits in type 1 BB/Wor-rats from 1 week to 10 month diabetes duration. Motor and sensory conduction velocities were decreased after 4 and 6 weeks of diabetes and declined further over the remaining 9 months. Myelinated sural nerve fibers showed progressive deficits in fiber numbers and sizes. Structural deficits in unmyelinated axonal size were evident at 2 month and deficits in number were present at 4 mo. These changes were preceded by decreased availability of insulin, C-peptide and IGF-1 and decreased expression of neurofilaments and *β*-III-tubulin. Upregulation of phosphorylating stress kinases like Cdk5, p-GSK-3*β*, and p42/44 resulted in increased phosphorylation of neurofilaments. Increasing activity of p-GSK-3*β* correlated with increasing phosphorylation of NFH, whereas decreasing Cdk5 correlated with diminishing phosphorylation of NFM. The data suggest that impaired neurotrophic support results in sequentially impaired synthesis and postranslational modifications of neuroskeletal proteins, resulting in progressive deficits in axonal function, maturation and size.

## 1. Introduction

Diabetic polyneuropathy (DPN) is a common complication in diabetic patients. Its pathogenesis is not fully understood and aspects of underlying mechanisms are controversial [1, 2]. Although hyperglycemia-induced metabolic abnormalities including polyol-pathway hyperactivity, oxidative stress, protein kinase C alteration and advanced glycation end-products contribute to type 1 DPN [3, 4], impaired insulin/C-peptide signaling has emerged as an additional initiating and important factor in its pathogenesis [2, 5]. 

 Apart from the direct neurotrophic effects exerted by insulin and C-peptide, they provide gene regulatory functions on other neurotrophic factors and their receptors [6, 7] with downstream effects on cytoskeletal and cell adhesive proteins and their postranslational modifications [8, 9]. Differences in insulin signaling-related effects are likely to underlie the differences between DPN in insulin-deficient type 1 and hyperinsulinemic type 2 diabetes [1, 10–14]. 

 The characteristic pathological features of DPN are axonal atrophy and nerve fiber loss, secondary to a dying back process. Early preceding functional deficits are believed to be metabolically induced by decreased Na^+^/K^+^-ATPase and eNOS activities precipitated by impaired insulin action and hyperglycemia [1–4, 13]. Axonal changes and nodal pathology are more severe in type 1 DPN, whereas primary segmental demyelination is characteristic of type 2 DPN [2, 8, 11]. In both cases though, DPN is a dynamic disease process with changing underlying causative pathobiological components. 

 Neurofilaments (NFs) and tubulins are major constituents of the axon cylinder and their expression levels and phosphorylation states determine axonal function, growth and caliber [13, 15–17]. Studies in animal models have demonstrated reduced expression of NFs and tubulins in dorsal root ganglion cells (DRGs), decreased axonal transport of NFs and aberrant phosphorylation of NFs in peripheral nerves [18–22]. Since NF mRNAs do not increase during development and radial axonal growth, postranscriptional regulations of NF appear to be more important. Several neurotrophic molecules, such as nerve growth factor (NGF), neurotrophin-3 (NT-3), insulin-like growth factor-1 (IGF-1), insulin and C-peptide stabilize NF transcripts [22, 23]. Aberrant phosphorylation of NFs perturbs their alignment and interaction with other cytoskeletal components resulting in impaired axonal function, and eventually atrophy and loss. Several kinases have been implicated in aberrant phosphorylation of NFs, such as cyclin dependent kinase 5 (Cdk5) and the MAP kinases Erk 1/2 (p44/42), stress-activated protein kinase/c-jun NH2-terminal kinase (SAPK/JNK) and phosphorylated glycogen synthase kinase 3*β* (p-GSK-3*β*) [21, 24, 25]. 

 Tubulins are other important cytoskeletal components assembling into microtubules, which provide a basis for axonal transport and polarity. Although the role of tubulins has not been fully explored in DPN, decreased mRNA and protein expression have been documented [18, 19, 26]. Microtubule-associated proteins such as MAP1B and 120 kD tau regulate the assembly and stability of microtubules in adult DRGs [27]. An additional protein that binds to the microtubule plus end is Atk-regulated GSK-*β*, where it regulates a number of microtubule binding proteins [28]. Inhibition of GSK-*β* by phosphorylation abolishes MAP1B phosphorylation and reduces microtubule stability and axonal outgrowth [29]. 

 Previous studies have emphasized the importance of cytoskeletal proteins for axonal function, whereas their relationship to axonal growth, pathology and nerve morphometry has not previously been examined longitudinally in experimental diabetes. Most studies have examined STZ-induced diabetic rats [18–22], a model in which structural changes in peripheral nerve are mild and which does not display progressive nerve fiber deficits. 

 The insulinopenic BB/Wor-rat, is a close model of human type 1 diabetes [30] and shows early structural changes followed by loss of myelinated and unmyelinated fibers as in the human condition [9, 31]. To explore the sequential and mechanistic role of neuroskeletal protein perturbations in the course of DPN development, diabetic BB/Wor-rats were investigated longitudinally from 1-week to 10-month duration of diabetes. We examined motor, sensory and C-fiber functions, quantitative structural abnormalities of myelinated and unmyelinated fibers in sural nerve, cytoskeletal protein expression, their phosphorylation as well as the expression of phosphorylating kinases in DRGs.

## 2. Materials and Methods

### 2.1. Animals

Male prediabetic BB/Wor-rats and age- and sex-matched nondiabetes prone BB-rats were obtained from Biomedical Research Models (Worcester, MA). Body weight, urine volume and glucosuria (Keto-Diastix, Bayer, Elkhart, IN) were monitored daily to ascertain onset of diabetes and for calibrating daily insulin doses. After onset of diabetes at 76 ± 4 days of age, diabetic rats received titrated doses (0.4–3.3 U/d) of protamine zinc insulin (Blue Ridge Pharmaceuticals, Greensboro, NC) to maintain constant blood glucose levels at approximately 25 mM and to prevent ketoacidosis. As previously demonstrated this paradigm maintains the animals well above (>15.0 mM) hypoglycemic levels [32]. Animals were cared for in accordance with the guidelines of the Animal Investigation Committee, Wayne State University and those of NIH (publication no. 85-23, 1995).

### 2.2. Thermal Plantar Test

Latencies of hind paw withdrawal to thermal stimulation (42°C; 152 mW/cm^2^) were used as measures of thermal algesia, reflecting unmyelinated fiber function, and were measured at 1-week, 1-week, 2-week, 4-week, 7-week and 10-month duration of diabetes. The measurements were performed using a UGO Biological Research Apparatus (Comerio, Italy). The time from heat source activation to the animal's self-withdrawal in seconds was measured six times in alternating hind paws. The mean of these measurements was calculated and used as the individual measure of latency withdrawal [33].

### 2.3. Electrophysiological Recordings

For nerve conduction velocities studies, animals were anesthetized with isoflurane inhalation (2-3%) mixed with oxygen delivered by an anesthesia machine system. Motor nerve conduction velocity (MNCV) and sensory nerve conduction velocity (SNCV) were measured in the left hind limb under temperature controlled (36-37°C) conditions using a Cadwell 5200A Electromyographer (Cadwell Laboratories, Kennewick, WA) as previously described in detail [8, 12, 26]. MNCV was measured at 1-week, 1-week, 2-week, 4-week, 7-week and 10-month duration of diabetes and SNCV at 6 week, 2-week, 4-week, 7-week and 10-month. 

### 2.4. Tissue Collection

After 2, 4, 7 and 10-month of diabetes, diabetic and age-matched control rats were sacrificed with an intraperitoneal overdose of sodium pentobarbital (120 mg/kg body weight). The right unifascicular sural nerve was dissected from five diabetic and five control rats at each time point and fixed in 1% cacodylate buffered (pH 7.4) 2.5% glutaraldehyde, dehydrated and embedded in Epon for morphometric analyses as previously described (6,35). Bilateral L4 and L5 DRGs from four animals per group at 2, 7, and 10-month of diabetes were collected for protein extraction. Tissues were snap frozen in liquid nitrogen and kept in −80°C until use. Animals were perfused with 500 mL of 0.1 M phosphate buffered (pH 7.4) 4% paraformaldehyde. L4 and L5 DRG's were postfixed in the same fixative, rinsed in PBS, dehydrated, immersed in xylene, embedded in paraffin and used for immunocytochemical studies.

### 2.5. Western Blotting

DRGs were lysed in detergent lysis buffer (50 mM Tris-HC1, pH 7.4, 150 mM NaCl, 1 mM EDTA, 1% Triton X-100, 1 mM phenylmethylsulfonyl fluoride, 1 *μ*g/mL leupeptin and 1 *μ*g/mL aprotinin). The lysates were centrifuged at 12,000 rpm for 20 minutes at 4°C and protein concentrations were measured using bicinchoninic acid protein assay reagent (Pierce, Rockford, IL) with bovine serum albumin as standard. Ten to 40 *μ*g of protein was separated by 7.5–10% SDS-PAGE, and transferred to PVDF membranes (Bio-Rad, Hercules, CA). Membranes were blocked with Tween-20-tris buffered saline (TTBS) (10 mM Tris-HC1, pH 7.5, 100 mM NaCl and 0.1% Tween 20) containing 5% nonfat dry milk (Bio-Rad, Hercules, CA) prior to incubation with primary antibodies. Primary antibodies were: Rabbit-polyclonal-antineurofilament heavy (C-terminal), rabbit-polyclonal-antineurofilament medium (C-terminal), rabbit- polyclonal-antineurofilament light, mouse-monoclonal-anti-*β*-III isoform of tubulin and mouse-monoclonal-antiactin were all purchased from Chemicon International (Temecula, CA). Mouse-anti-*β* tubulin was from Oncogene Research (Cambridge, MA). Rabbit antiphospho tau antibodies (PS 396 and PS 404) were gifts from Dr. K. Ishiguro, Mitsubishi Kagaku Institute of Life Sciences, Tokyo, Japan. Mouse-monoclonal-antineurofilament-phosphorylataed epitope (SMI31) was from Covance Research Products (Berkeley, CA), rabbit-polyclonal-phosphorylated p44/42 MAP kinase (Thr202/Tyr204), rabbit-polyclonal-total p44/42 MAP kinase, mouse-monoclonal-antiphosphorylated SAPK/JNK (Tyr180/Tyr182) and rabbit monoclonal antiphospho p38 were from Cell Signaling Technology (Danvers, MA). Goat-polyclonal-anti-GSK-3*β* and p-GSK-3*α*/*β* was from Santa Cruz Biotechnology (Santa Cruz, CA), and rabbit-polyclonal-anti-Cdk5 was from Abcom Inc. (Cambridge, MA). Antigen detection was performed using chemiluminescence (Amersham Pharmacia Biotech, Piscataway, NJ) with horseradish peroxidase-conjugated secondary antibodies. Membranes were exposed to Biomax film (Kodak, Rochester, NY). Images were scanned and densities determined by a Bio-Rad Fluoro-S multimager (Bio-Rad, Hercules, CA). Expression of proteins was corrected for by actin density and expression in control animals was arbitrarily set to 1.0.

### 2.6. Morphometry

Semithin (0.5 *μ*m) cross-sections of Epon-embedded sural nerves were stained with toluidine-blue for light microscopic morphometric analysis using a computerized image analysis system (Image-1, Universal Imaging Corp, West Chester, PA). This system is programmed to assess the total complement of sural nerve myelinated fibers and provides the following parameters: number of fibers (#), fiber density (#/mm^2^), mean fiber area (*μ*m^2^), mean axonal area (*μ*m^2^), mean myelin area (*μ*m^2^), axon/myelin ratio, index of circularity and fiber occupancy rate (% of total fascicular area) as previously described [34]. 

 For morphometric analyses of unmyelinated fibers, ultrathin cross sections of sural nerves were obtained from a LKB ultramicrotome (Marviac Limited, Halifax, Canada) and stained with uranyl acetate and lead citrate. They were examined in a Zeiss EM 900 electron microscope (Carl Zeiss, Oberkochen, Germany). Systematically selected frames representing 25 to 30% of the sural nerve cross-sectional area were obtained. Photographs were enlarged 10,000 times and downloaded to the image analysis system. The following morphometric parameters of unmyelinated fibers were obtained: unmyelinated fiber number, fiber density (#/mm^2^), mean fiber size (*μ*m^2^), axon numbers per Schwann cell unit and the frequencies of collagen pockets, denervated Schwann cell profiles, type 2 axon-Schwann cell relationship (disruption of the mesaxon) and regenerating C-fibers [33].

### 2.7. Immunostaining

Deparaffinized 6 *μ*m sections of DRGs were incubated with selected primary antibodies (Cdk5, GSK-3*β* and SMI-31, same sources as above) for 30 minutes at room temperature, washed with three changes of PBS, and incubated with peroxidase-conjugated secondary antibodies (Vector Lab, Burlingame, CA) for 30 minutes at room temperature. The immunoreactive products were visualized with DAB as color chromogen.

### 2.8. Statistical Analysis

All values were expressed as means ± SD. Significance of differences was analyzed by ANOVA. Group differences were assessed by Scheffes test. Significance was defined as a *P*-value less than .05. To assess the relationship between expression of stress-kinases and hyperphosphorylation of individual neurofilaments linear or logistic regression analyses were performed. All analyses were performed by personnel unaware of the animal identities.

## 3. Results

### 3.1. Clinical Findings

Blood glucose and glycated hemoglobin levels were consistently and significantly (*P* < .001) elevated in diabetic rats. Plasma insulin levels were markedly reduced (*P* < .001) throughout the observation period. As a reflection of pancreatic *β*-cell loss, C-peptide levels were severely decreased or not measurable. Plasma IGF-1 levels were significantly decreased already in 2-month diabetic rats (*P* < .001) and remained decreased compared to control values (Table 1).

### 3.2. Latencies to Thermal Stimulations

**Figure 1:**
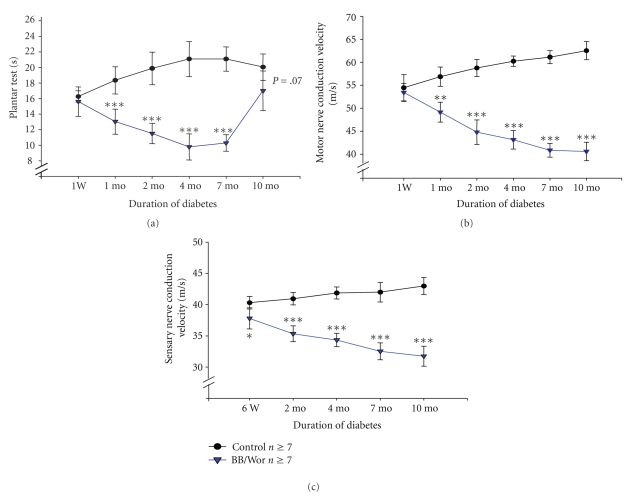
(a) Latencies of thermal plantar tests of control and diabetic BB/Wor-rats at 1-week, 1-month, 2-month, 4-month, 7-month and 10-month duration. (b) Motor nerve conduction velocity of control and diabetic BB/Wor-rats at 1-week, 1-week, 2-week, 4-week, 7-week and 10-month duration; (c) Sensory nerve conduction velocity of control and diabetic BB/Wor-rats at 6 week, 2-week, 4-week, 7-week and 10-month duration. Results are means ± SD (*n* = 8) *P < .05, **P < .01 and ***P < .001 versus respective control values.

Hyperalgesia, as reflected by decreased withdrawal latencies, was significantly (*P* < .001) altered at 1-month of diabetes. Latencies decreased up to 4-month of diabetes, then plateaud and increased (*P* < .001) from 7 to 10-month of diabetes, at which time it was not different (*P* = .07) from that of control animals (Figure 1(a)). The increase in latencies from 7 to 10-month most likely represent increasing hypoalgesia due to progressive C-fiber deficits (see below). 

### 3.3. Motor Nerve Conduction Velocity (MNCV)

MNCV was normal at one-week of diabetes, however, it was significantly (*P* < .05) decreased at one month and decreased progressively thereafter to reach 68% (*P* < .001) of normal values at 10-month of diabetes (Figure 1(b)).

### 3.4. Sensory Nerve Conduction Velocity (SNCV)

Only after 6 wks of diabetes did diabetic rats show a significantly (*P* < .05) reduced SNCV, a deficit that increased progressively with duration of diabetes to reach 78% (*P* < .001) of normal values at 10-month (Figure 1(c)).

### 3.5. Myelinated Fiber Morphometry of the Sural Nerve (Table 3)

**Figure 2:**
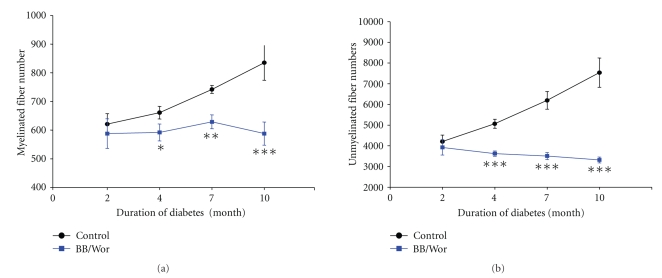
Longitudinal morphometric data showing myelinated and unmyelinated fiber numbers. Note increasing deficits in both myelinated and unmyelinated fibers in diabetic rats compared to age-matched control rats. However the absolute fiber numbers in diabetic rats did not change over the observation period, suggesting a component of impaired fiber maturation in diabetic animals. *P < .05, **P < .01, ***P < .001 versus age-matched control values.

Only a slight decrease (*P* < .05) in the circularity of myelinated axons was evident at 2-month of diabetes. Four month diabetic rats showed a significant deficit in axonal size (*P* < .005) of myelinated fibers (Table 2). After 7-month of diabetes, there was a robust difference (*P* < .001) in fiber size due to axonal (*P* < .001) and myelin (*P* < .005) atrophy compared to control rats, which was reflected in a further decrease in the index of circularity (*P* < .001) (Table 2). The most striking change though was a 15% (*P* < .005) deficit in myelinated fiber number. This progressed to 30% (*P* < .001) at 10-month of diabetes (Figure 2(a)), which was reflected in a decreased (*P* < .05) occupancy rate (Table 2). 

### 3.6. Unmyelinated Fiber Morphometry

Two month diabetic animals showed mild (*P* < .05) growth deficits of unmyelinated fibers which became more pronounced to reach 72% (*P* < .005) of normal values at 10-month of diabetes (Table 3). Moreover there were significant deficits in unmyelinated fiber numbers from 4-month of diabetes onwards (*P* < .001), which amounted to 56% (*P* < .001) at 10-month of diabetes (Figure 2(b)). These deficits were preceded and accompanied by significant degenerative changes, such as increased frequencies of type 2 Schwann cell/axon relationship (disruption of the mesaxon) (*P* < .001), collagen pockets (*P* < .01) and denervated Schwann cell profiles (*P* < .001). Concomitant with ongoing degeneration, diabetic rats showed increased rates of regenerating C-fibers (Table 3).

### 3.7. Expression of NFH, NFM and NFL and Phosphorylation of NFH and NFM

**Figure 3:**
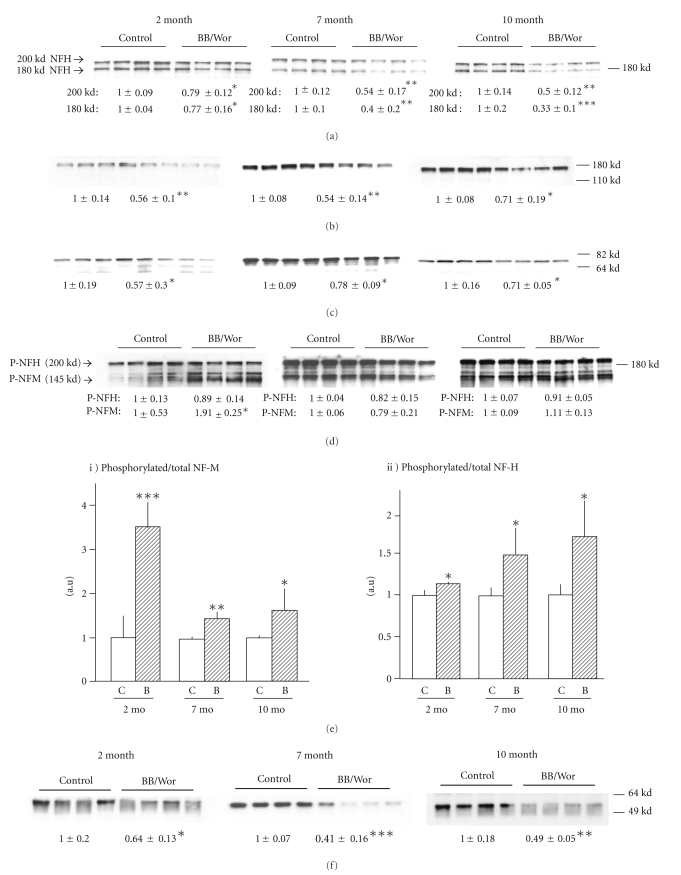

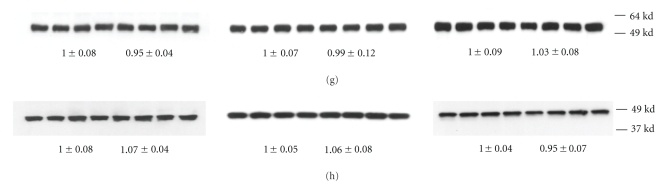
Levels of phosphorylated and total neurofilament subunits in DRGs. (a) total NFH; (b) total NFM; (c) total NFL; (d) SMI31 (phosphorylated NFM and NFH); and (e) Relative phosphorylation of NFM (ei) and NFH (eii) at 2-month, 7-month and 10-month of diabetes. Results are means ± SD (*n* = 4). *P < .05, **P < .005 and ***P < .001 versus respective control rats. Expression of DRG tubulins. (f) betaIII-tubulin, (g) total beta-tubulin and (h) actin levels in DRGs at 2-month, 7-month and 10-month diabetic rats. Results are means ± SD (*n* = 4) *P < .05, **P < .005 and ***P < .001 versus respective control rats.

NFH expression showed two major bands at molecular weights 180 kd and 200 kd. In 2, 7 and 10-month diabetic rats NF-H expression in DRGs of both 180 kd (*P* < .05, *P* < .005 and *P* < .001, resp.) and 200 kd (*P* < .05, *P* < .005 and *P* < .005, resp.) were significantly and progressively decreased (Figure 3(a)). The decreased expression of NF-M at 2-month of diabetes remained relatively constant over the 10-month course (*P* < .005, *P* < .005 and *P* < .05 resp.) (Figure 3(b)). The decreased expression of NF-L also remained constant (*P* < .05, *P* < .05 and *P* < .05) in diabetic DRGs at 2, 7 and 10-months respectively (Figure 3(c)). With the specific antibody, SMI 31, phosphorylated NFH and NFM were detected. There was no absolute change in phosphorylated NFH during the observation period, whereas a doubling (*P* < .05) of phosphorylated NFM occurred at 2-month of diabetes and then declined (Figure 3(d)). On the other hand, the proportion of phosphorylated NFM relative to NFM expression was increased in 2-month (*P* < .001), 7-month (*P* < .005) and 10-month (*P* < .05) diabetic rats (Figure 3(e)i). Therefore, the proportion of phosphorylated NFM decreased with duration of diabetes. The ratios of phosphorylated NFH, calculated as the proportion of total NFH expression, were significantly increased in 2, 7 and 10-months of diabetic rats (*P* < .05) and tended to increase with duration of diabetes (Figure 3(e)ii). The expression of phosphorylated tau (PS 396 and PS 404) was not altered in diabetic rats for any of the epitopes (data not shown).

### 3.8. Expression of Tubulin in DRGs

Neuron specific *β*III-tubulin levels were reduced in diabetic animals at 2, 7, and 10-month (*P* < .05, *P* < .001, *P* < .005, resp.) (Figure 3(f)), whereas total *β*-tubulin levels were not altered at any time point (Figure 3(g)).

### 3.9. Phosphorylation of MAPKs in DRGs

**Figure 4:**
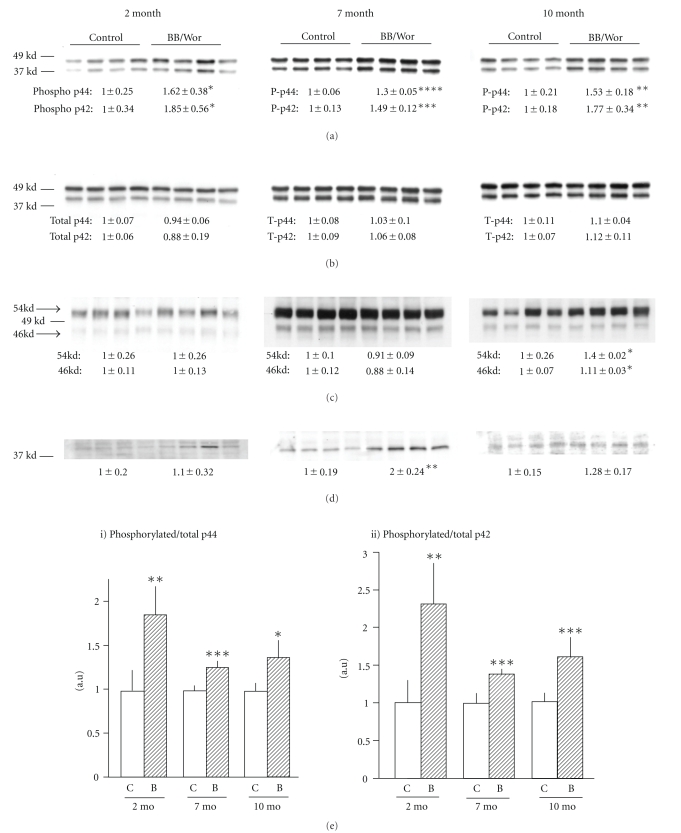
Protein expressions of activated kinases: (a) phosphorylated p42/44; (b) total p42/44; (c) phosphorylatead SAPK/JNK;. (d) phosphorylated-p38; (e) Ratio of phosphorylated / total p44 and p42 at 2-month, 7-month and 10-month duration. Phophorylation ratio was calculated by dividing phosphorylated protein level with respective total protein level. Results are means ± SD (*n* = 4). *P < .05, **P < .01, ***P < .005 and ****P < .001 versus respective control rats.

There were significant increases in phosphorylated p42 (Erk 2) in DRGs at 2, 7 and 10-months of diabetes (*P* < .05, *P* < .005 and *P* < .01, resp.) and phosphorylated p44 (Erk 1) (*P* < .05, *P* < .001 and *P* < .01, respectively) (Figure 4(a)), whereas total p42 and p44 levels did not change (Figure 4(b)). The proportions of phosphorylated p44 (*P* < .01, *P* < .005 and *P* < .05, resp.) and p42 (*P* < .01, *P* < .005 and *P* < .005, resp.) were therefore increased in 2,7 and 10-month diabetic rats (Figures 4(e)i and 4(e)ii). Both 46 kd and 54 kd phosphorylated SAPK/JNK levels in DRGs were unchanged in 2 and 7-month diabetic rats, but significantly increased in 10-month diabetic rats (both *P* < .05) (Figure 4(c)). Phosphorylated p38 was not altered in 2-month diabetic rats, but increased in 7-month diabetic rats (*P* < .01) and returned to normal at 10-month (Figure 4(d)).

### 3.10. p-GSK-3*β* and Cdk 5 in DRGs

**Figure 5:**
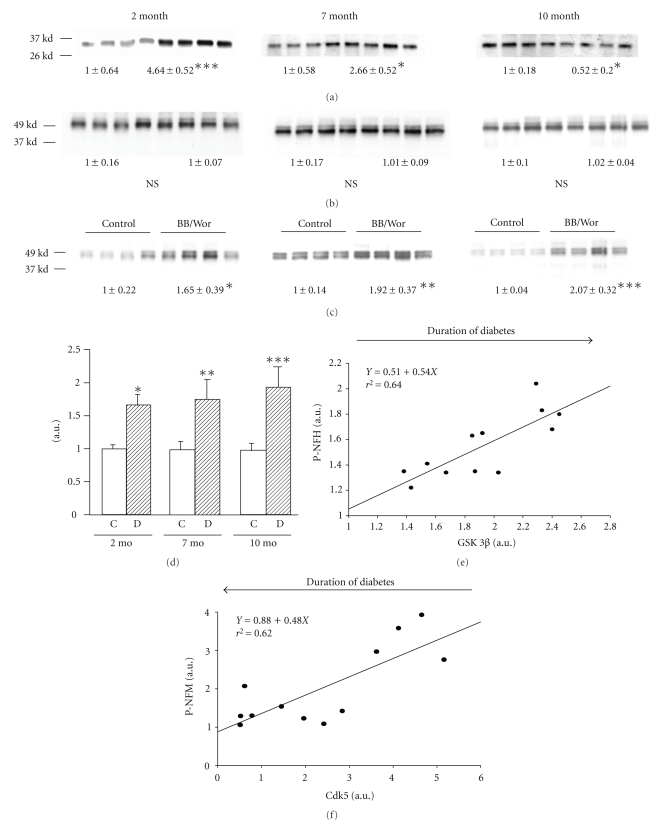
Expression of Cdk5 (a), total GSK-3*β* (b) and phosphorylated GSK-3*β* (c) in control and diabetic BB/Wor-rats at 2-month, 7-month, and 10-month duration. Results are means ± SD (*n* = 4). *P < .05, **P < .01, ***P < .001 versus respective control rats. Linear regression analyses of the expression of GSK-3*β* and relative expression of phosphorylated NFH (e) and of the expression of Cdk5 and relative phosphorylated NFM (f). Note increased expression of GSK-3*β* and phosphorylated NFH with duration of diabetes, whereas decreased expression of Cdk5 correlated with decreased phosphorylation of NFM with increasing diabetes duration.

Cdk5 showed a more than four-fold increase (*P* < .001) in 2-month diabetic rats and then declined at 7-month (*P* < .05) and declined further to below control values (*P* < .05) at 10-month (Figure 5(a)). Total GSK-3*β* was not altered throughout the observation period (Figure 5(b)), whereas p-GSK-3*β* increased gradually with duration of diabetes (Figure 5(c)). It was modestly increased (*P* < .05) in 2-month diabetic rats, but increased further at 7 (*P* < .01) and 10-month (*P* < .001) duration of diabetes (Figure 5(a)). This resulted in an increasing proportion of GSK-3*β* being phosphorylated (Figure 5(d)). p-GSK-3*β* correlated linearly with increasing phosphorylation of NFH (*P* < .002) (Figure 5(e)). Significant correlation (*P* < .002) existed between decreasing expression of Cdk5 and the decreasing proportion of phosphorylated NFM over the course of diabetes (Figure 5(f)).

### 3.11. Immunohistochemistry

**Figure 6:**
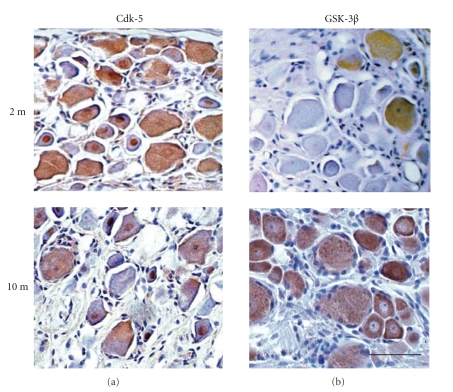
Cdk5 (a) and p-GSK-3*β* (b) positive DRG neurons in 2-month and 10-month diabetic rats. Note frequent Cdk5 stainability of small DRG neurons at 2-month (a), which tended to decrease with duration of diabetes. The number of p-GSK-3*β* positive DRG neurons increased with duration of diabetes (b) and affected ganglion cells of all sizes. The bar in the lower Figure 5(b) equals 35 *μ*m and indicates the magnification of all frames.

Immunostaining for Cdk5 showed frequent positive staining of small DGR neurons and their nuclei in 2-month diabetic rats, which decreased with duration of diabetes (Figure 6(a)). Only sporadic GSK-3*β*-positive neurons were present in 2-month diabetic. In 10-month diabetics there was a general positivity of both small and large DRG neurons (Figure 6(b)).

## 4. Discussion

Here we show that early deficits in insulin and C-peptide availability and decreased presence of IGF-1 as well as hyperglycemia are associated with impaired functions of unmyelinated and myelinated nerve fibers. Such changes are accompanied in DRGs by perturbations of the IGF-1 and insulin receptors, transcription factors such as NF-*κ*B and early gene response factors such as c-fos perpetuating impaired protein expression of neurofilaments and *β*-III tubulin [7, 26, 35, 36]. Consequent decreases in insulin and IGF-1 signaling activities enhance the expression of so-called stress kinases such as p-GSK-3*β*, p42/44, Cdk5 and SAPK/JNK with affinities to cytoskeletal elements [37]. Resulting malalignment and disassembly of interactive neurofilaments and tubulins result in progressively impaired axonal growth, atrophy and eventually axon loss. This sequence of events appears to affect unmyelinated fibers earlier and more severely than myelinated fibers paralleling the early impaired neurotrophic support by insulin and NGF, which target specifically small DRG neurons [19, 33, 38, 39]. 

 The initial functional deficits of unmyelinated fibers are most likely due to hyperexcitability of C-fibers through increased activation of tetrodotoxin-resistant Na-channels [40, 41]. In myelinated fibers the early and reversible nerve conduction deficits have been related to insulin deficiency- and hyperglycemia-related suppression of nodal Na^+^/K^+^-ATPase activities [1, 3, 8]. 

 As shown previously in this model, impaired availability of insulin and C-peptide affects the gene expression of IGF-1, NGF and NT-3 and their receptors [23, 36, 42] with consequences for the expression of neuroskeletal proteins [43, 44]. NFs are unique to neurons and interact with microtubules and other cytoskeletal elements and form the basis for axonal transport. NFs consist of three intermediate filament proteins, NFL (61 kD), NFM (90 kD) and NFH (110 kD). They have a central domain in common involved in the formation of coiled-coil dimers. Two dimers align in a staggered fashion to form tetramer and eventually 10 nm filaments [15]. The formation of NF heteropolymers requires NFL to aggregate with either NFM or NFH [45]. NFL and NFM are coordinately expressed suggesting the same transcriptional signals, whereas NFH is regulated separately [46]. During their assembly in the perikaryon, NFL acts as the core and NFM and NFH become phosphorylated as the coil leaves the perikaryon, important for the formation of cross-bridges, intrafilament spacing, axonal growth and conduction velocity [47]. 

 Here we demonstrate marked suppression in the coordinately expressed NFL and NFM at 2-month, which remained constant, whereas NFH expression declined with duration of diabetes. Initially, 78% of NFM showed aberrant phosphorylation, which decreased with duration of diabetes. On the other hand the proportion of phosphorylated NFH increased with diabetes duration to reach 1.7-fold at 10-months duration. These findings indicate that impaired assembly and function of NFs occur early in diabetic neuropathy initiated by aberrant phosphorylation of NFM followed by increasing phosphorylation of NFH. The aberrant phosphorylation can be correlated with generation of stress-kinases secondary to impaired insulin and IGF-1 signaling. These changes alone are likely to perturb the interactions with tubulins. Several stress-related kinases have been identified as being responsible for aberrant neurofilament phosphorylation [21]. The early increase in p35 regulated Cdk5 is likely to underlie early phosphorylation of specific neurofilament side arm fragments [48], as indicated by the decreasing expression of Cdk5 with duration of diabetes paralleling decreasing phosphorylation of NF-M. Immunoreactivity of Cdk5 in 2-month diabetic rats involved mainly small nociceptive DRG ganglion cells suggesting that this neuronal population is affected early as indicated by the morphometric analyses of C-fibers and previous findings of early deficits of small nociceptive neurons [33]. Over-activity of the p35/Cdk5 complex has also been associated with hyperglycemia and has been implicated in the hyperphosphorylation of MAP and tau in Alzheimer's disease [49]. Interestingly, phospho-tau was not significantly altered in diabetic DRG's. On the other hand, p-GSK-3*β* expression increased with duration of diabetes in parallel with decreased Akt-mediated phosphorylation of GSK-3 at Ser 9 [50, 51] and correlated with increased phosphorylation of NF-H reflecting its affinity to NF-H [48]. Hence, impaired insulin and IGF-1 signaling resulting in decreased GSK-3*β* (Ser 9) expression allows for phosphorylation of p-GSK-3*β*, a potent phosphorylating stress kinase, which has also been implicated in Alzheimer-like changes in diabetes [51]. Differential phosphorylation of neurofilaments by Cdk5 and p-GSK-3*β* is known to occur [48, 52]. Additional potential phosphorylating kinases demonstrated here are p42/44 originating from deprived MAPK signaling. The expression of phosphorylated p42/44 remained relatively constant throughout the observation period, whereas the 54-kDA isoform of JNK was increased only after 10-month of diabetes, which is in agreement with the findings by Fernyhough et al. [21]. This is also consistent with increased phosphorylation of NF-H at this time point, since it provides a substrate for activated JNK [53]. Activation of JNK has been associated with NGF and insulin withdrawal [7, 54]. Demonstrated here for the first time several kinases become activated at different stages of diabetes and show affinities to specific neurofilament subunits whose expression also varies with diabetes duration resulting in a dynamic picture of aberrant phosphorylation changes with duration of diabetes. 

 As shown here *β*-III tubulin expression is severely diminished in acutely diabetic DRG neurons. Microtubules are assembled from *α* and *β* tubulin heterodimers, organized in a polarized fashion with a “minus” and “plus” end, at which polymerization of tubulin dimers occurs [29]. The present findings of decreased expression of *β*-III-tubulin therefore suggest perturbed polymerization of tubulin dimers. Decreased GSK-3*β* by impaired insulin and IGF-1-signaling is likely to inhibit MAP1B phosphorylation and hence further inhibit the stability and assembly of tubulins essential for axonal transport [27]. It therefore appears clear that multiple aspects of expression and postranslational events determining normal assembly of neuroskeletal constituents are severely altered already during the acute stage of DPN leading to impaired axonal maturation and degeneration. The sequential phosphorylation of NF components as well as destabilization of microtubules by several stress kinases can be traced to impaired neurotrophic signaling activities including those of insulin itself. 

 This notion is supported by the findings that replacement of insulinomimetic C-peptide, which normalizes insulin signaling, corrects the expression of neurotrophic factors, their receptors and neuroskeletal proteins as well as their postranslational phosphorylation [23, 26, 33]. These effects translate into improved function and amelioration of impaired maturation, axonal atrophy and loss [23, 34]. This construct may explain the fact that hyperinsulinemia in the type 2 BBZDR/Wor-rat causes substantially milder effects on neurotrophic factors and their receptors [9, 36] with significantly milder functional and axonal structural deficits [2, 9]. The changing and dynamic perturbations of the integrity of neuroskeletal proteins inevitably contribute to axonal dysfunction and malalignment of neuroskeletal components as previously demonstrated ultra-structurally [55], eventually resulting in axonal atrophy and progressive dying back of nerve fibers as shown here. 

 Apart from progressive degenerative changes, an additional componet of neuropathy in type 1 diabetes, not previously considered, is the potential impact of the perturbations demonstrated here on peripheral nerve maturation. As shown here, myelinated and unmyelinated fiber numbers and sizes did not change in absolute values in diabetic animals between 2 and 10-month, whereas both parameters increased significantly during the same time span in control rats. In the rat peripheral nerve maturation is only completed at 6 month [56] and the corresponding age in humans is beyond 25 years of age [57]. This suggests that metabolic perturbations interfering with normal maturation processes are likely to retard nerve growth and contribute to diabetic neuropathy both in humans and murine models, as has been suggested for diabetic encephalopathy accompanying type 1 diabetes. 

 In summary, the present findings strongly suggest that insulin and C-peptide deficiencies in type 1 DPN sets into motion a dynamic cascade of events leading to suppression of other neurotrophic factors together resulting in impaired expression of neuroskeletal constituents. Simultaneous activation of stress-kinases leads sequentially to their aberrant phosphorylation compromising axonal function, and promoting progressive axonal growth deficits, atrophy and loss, the very hallmark of DPN.

## Figures and Tables

**Table 1:** Clinical data.

		N	Body weight (g)	Blood glucose level (mM)	Insulin pmol/L	C-peptide pmol/L	IGF-1 ng/mL
2-month	Control	8	428 ± 19	5.0 ± 0.4	455 ± 52	710 ± 52	1785 ± 112
	BB/Wor	8	348 ± 21*	24.6 ± 2.2*	57 ± 7*	43 ± 12*	1328 ± 87*
4-month	Control	8	429 ± 22	5.0 ± 0.3	438 ± 27	723 ± 12*	—
	BB/Wor	8	375 ± 15^†^	23.9 ± 1.3*	51 ± 6*	<25*	—
7-month	Control	9	505 ± 37	5.3 ± 0.3	526 ± 108	727 ± 24	1182 ± 32
	BB/Wor	9	370 ± 18*	25.2 ± 1.7*	153 ± 55*	<25*	778 ± 70*
10-month	Control	8	555 ± 29	5.0 ± 0.3	506 ± 81	687 ± 18	1192 ± 37
	BB/Wor	8	400 ± 13*	23.2 ± 1.3*	84 ± 21*	<25*	747 ± 86*

Results are means ± SD. ^†^ *P* < .01; *P < .001 versus respective control-rats.

**Table 2:** Myelinated fiber morphometry.

	2-month control	2-month BB/Wor	4-month control	4-month BB/Wor	7-month control	7-month BB/Wor	10-month control	10-month BB/Wor
Fiber area (*μ*m^2^)	30.9 ± 2.6	30.7 ± 3.3	36.2 ± 3.8	32.5 ± 3.6	41.7 ± 1.0	33.3 ± 2.3****	38.9 ± 2.0	31.4 ± 1.5****
Axon area (*μ*m^2^)	11.7 ± 1.0	11.5 ± 1.9	15.0 ± 1.6	11.9 ± 1.4***	18.5 ± 0.5	13.6 ± 1.4****	16.2 ± 1.1	12.4 ± 0.8****
Myelin area (*μ*m^2^)	19.2 ± 2.0	19.2 ± 1.8	22.1 ± 2.4	20.4 ± 1.3	24.8 ± 2.1	19.7 ± 1.6***	22.7 ± 1.7	19.0 ± 1.5**
a/m ratio	0.613 ± 0.063	0.601 ± 0.079	0.655 ± 0.119	0.624 ± 0.084	0.750 ± 0.065	0.692 ± 0.079	0.716 ± 0.069	0.654 ± 0.071
OR (%)	49.8 ± 7.6	46.6± 4.2	49.7 ± 3.5	52.4± 3.6	61.3 ± 3.7	58.3 ± 1.8	57.9 ± 4.7	50.0 ± 5.8*
I.C.	0.955 ± 0.012	0.926 ± 0.023*	0.937 ± 0.013	0.906 ± 0.017	0.946 ± 0.005	0.900 ± 0.007****	0.924 ± 0.011	0.895 ± 0.026*

Results are means ± SD. *P < .05, **P < .01, ***P < .005 and ****P < .001 versus respective control rats.

**Table 3:** Unmyelinated fiber morphometry.

	2-month control	2-month BB/Wor	4-month control	4-month BB/Wor	7-month control	7-month BB/Wor	10-month control	10-month BB/Wor
Axon number/SC (#/unit)	5.01 ± 0.81	4.47 ± 0.54	5.26 ± 0.64	4.22 ± 0.29*	5.29 ± 0.26	4.17 ± 0.11****	5.20 ± 0.34	4.03 ± 0.26****
Mean fiber area (*μ*m^2^)	0.636 ± 0.076	0.534 ± 0.049*	0.742 ± 0.090	0.613 ± 0.042*	0.706 ± 0.072	0.531 ± 0.044***	0.676 ± 0.056	0.487 ± 0.076***
Denervated SC (%)	0.08 ± 0.11	0.86 ± 0.40***	0.24 ± 0.18	0.97 ± 0.26***	1.00 ± 0.32	2.61 ± 0.57****	2.54 ± 0.90	4.42 ± 1.32*
Collagen pocket (%)	0.76 ± 0.35	1.95 ± 0.78*	0.62 ± 0.42	1.59 ± 0.57*	1.65 ± 0.32	3.15 ± 0.89**	7.98 ± 0.49	11.60 ± 3.36*
Type 2 relationship (%)	2.21 ± 0.40	4.29 ± 0.91***	1.18 ± 0.13	6.45 ± 2.00****	3.30 ± 0.54	14.45 ± 3.02****	6.04 ± 1.47	18.22 ± 2.36****
Regenerating fiber (%)	0.84 ± 0.39	1.59 ± 0.32**	0.56 ± 0.43	2.07 ± 0.36****	1.69 ± 0.51	2.49 ± 0.46*	4.28 ± 1.24	8.56 ± 3.13*

Results are means ± SD. *P < .05; **P < .01; ***P < .005; and ****P < .001
